# Nonuniformity correction algorithm with efficient pixel offset estimation for infrared focal plane arrays

**DOI:** 10.1186/s40064-016-3534-1

**Published:** 2016-10-21

**Authors:** Tomasz Orżanowski

**Affiliations:** Institute of Optoelectronics, Military University of Technology, 2 Kaliskiego St., 00-908 Warsaw, Poland

**Keywords:** Infrared focal plane array, Nonuniformity correction, Fixed-pattern noise

## Abstract

This paper presents an infrared focal plane array (IRFPA) response nonuniformity correction (NUC) algorithm which is easy to implement by hardware. The proposed NUC algorithm is based on the linear correction scheme with the useful method of pixel offset correction coefficients update. The new approach to IRFPA response nonuniformity correction consists in the use of pixel response change determined at the actual operating conditions in relation to the reference ones by means of shutter to compensate a pixel offset temporal drift. Moreover, it permits to remove any optics shading effect in the output image as well. To show efficiency of the proposed NUC algorithm some test results for microbolometer IRFPA are presented.

## Background

Infrared focal plane arrays are widely used in various military and civil systems for thermal imaging. However they suffer from pixel-to-pixel responsivity (gain) and offset variations which induce a spatial noise called a fixed-pattern noise (FPN) in the image obtained from the detector array (Mooney et al. 1989). For instance, cooled HgCdTe IRFPAs offer the high electro-optical performance at the operating temperature of 77 K but for the long wavelength infrared (LWIR) region they exhibit a higher response nonuniformity than type-II InAs/GaInSb superlattice structures or quantum well infrared photoconductors (QWIPs) (Rogalski 2011). Modern uncooled microbolometer IRFPAs attain high performance and they become a good choice for cost-effective thermal imaging systems operating in LWIR range (Trouilleau et al. 2009). However they need some additional compensation due to inherent temporal drift of detector characteristics and the impact of housing temperature change on the detector array response. In order to obtain high thermal resolution of the infrared imagery, the IRFPA response nonuniformity must be reduced an order of magnitude below pixel temporal noise (Mooney and Shepherd 1996). For instance, to get a thermal resolution of 20 mK in the system operating in LWIR region where the scene contrast is about 2 %/K, the detector array response nonuniformity must be <0.04 % (*σ*/*m*) (Rogalski 2011).

Typical IRFPA response nonuniformity correction (NUC) relies on the signal processing of detector array output in order to remove FPN from the obtained image. In general, NUC methods are divided on reference-based and scene-based ones. The former use extended surface IR references as the uniform temperature sources to determine the suitable correction coefficients (Orżanowski and Madura 2010). The latter are reference-free and the coefficients for detector signal correction are obtained by the statistical analysis of pixel response in real-scene image sequences acquired by the thermal camera (Hayat et al. 1999). The integration of reference-based and scene-based technique into the radiometrically accurate NUC algorithm for IRFPA sensors is presented in the paper by Ratliff et al. (2003). The scene-based NUC methods are more sophisticated and they need the special operations to reduce “ghosting” artifacts appearing in the image after correction when the observed scene gives strong edges or slow global motion (Rossi et al. 2010).

The commonly used reference-based NUC method is the linear two-point calibration (TPC) (Perry and Dereniak 1993). The TPC algorithm is well known and it allows to compensate both gain and offset variations of particular pixels in the array. Moreover, it is easy to implement by hardware and quite sufficient in many applications. Even though this basic NUC algorithm is elaborated in detail, the efficient method of correction coefficients update, especially for pixel offsets, is dissembled or the one-point calibration by means of IR reference is suggested only.

In this paper a modified TPC algorithm enabling pixel offset correction coefficients update and removing optics shading effect by the proper usage of temporally averaged IRFPA response determined at closed inner shutter is presented. Since the IRFPA response on infrared radiance coming from the inner shutter does not include the impact of camera housing and optics infrared radiance then the direct using of that detector response as the offset correction coefficients can lead to the insufficient NUC results appearing as shading effect on the output image. In the presented correction scheme, the pixel response change determined at the actual operating conditions in relation to the reference ones at closed shutter is used to compensate a pixel offset temporal drift. It will be shown further that the proposed NUC algorithm offers some advantages in signal processing path and hardware implementation.

## Proposed NUC algorithm

The commonly used TPC algorithm is given by (Perry and Dereniak 1993)

1 $$ y_{i} \left( \phi \right) = \left[ {x_{i} \left( \phi \right) - x_{i} \left( {\phi_{1} } \right)} \right]g_{i} + x\left( {\phi_{1} } \right) $$

where *y*_*i*_(*ϕ*) and *x*_*i*_(*ϕ*) are corrected and uncorrected response of *i*th pixel in the array, respectively, *x*_*i*_(*ϕ*_1_) is the individual pixel response and *x*(*ϕ*_1_) is the average pixel response in the array at first calibration point *ϕ*_1_, *g*_*i*_ is the pixel gain correction coefficient. Since subtraction *x*_*i*_(*ϕ*) − *x*_*i*_(*ϕ*_1_) results in pixel offset equalization in the array then *x*_*i*_(*ϕ*_1_) term in Eq. 1 is considered as the required pixel offset correction coefficient. Accordingly, the *x*(*ϕ*_1_) term in Eq. 1 determines a global pixel offset value in the array. The pixel gain correction coefficient, *g*_*i*_, is defined as a ratio of the average pixel response change to the individual pixel response change within irradiance flux range from *ϕ*_1_ to *ϕ*_2_ and it is given by

2 $$ g_{i} = \frac{{\Delta x\left( \phi \right)}}{{\Delta x_{i} \left( \phi \right)}} = \frac{{x\left( {\phi_{2} } \right) - x\left( {\phi_{1} } \right)}}{{x_{i} \left( {\phi_{2} } \right) - x_{i} \left( {\phi_{1} } \right)}} $$

where *x*_*i*_(*ϕ*_1_) and *x*_*i*_(*ϕ*_2_) are temporally averaged responses of *i*th pixel in the array at first and second calibration point, respectively, *x*(*ϕ*_1_) and *x*(*ϕ*_2_) are spatially averaged array responses corresponding to the calibration points. Temporal averaging of the reference image sequences helps to reduce the impact of random noise (Ratliff et al. 2003). In order to update offset correction coefficients, *x*_*i*_(*ϕ*_1_), the calibration by means of shutter as the IR reference is performed. The temporally averaged IRFPA response obtained at closed shutter is used as the new table of pixel offset correction coefficients in TPC algorithm according to Eq. 1. It is important to take the shutter image sequence between the calibration points. This approach to update of offset correction coefficients provides efficient NUC performance when the external shutter rather than internal one is applied. The internal shutter does not include entire optics path and the computed offset correction coefficients are not accurate. As a result the optics shading effect in the image after correction is observed (see Fig. 2a). Hence, it is needed to modify the conventional TPC algorithm in order to decrease this image shading effect and to provide efficient method of offset correction coefficients update.

Let the pixel response change determined at closed shutter be defined as

3 $$ \Delta s_{i} \left( \phi \right) = s_{i} \left( \phi \right) - s_{i} \left( {\phi_{r} } \right) $$

where *s*_*i*_(*ϕ*) and *s*_*i*_(*ϕ*_*r*_) are temporally averaged responses of *i*th pixel in the array on incident irradiance flux, *ϕ*, obtained with closed shutter at actual and reference operating conditions, respectively. Since pixel response change, Δ*s*_*i*_(*ϕ*), is related to the pixel offset drift value then it can be introduced directly into the basic TPC algorithm formula as follows

4 $$ y_{i} \left( \phi \right) = \left[ {x_{i} \left( \phi \right) - x_{i} \left( {\phi_{1} } \right) -\Delta s_{i} \left( \phi \right)} \right]g_{i} + x\left( {\phi_{1} } \right) + \left[ {s\left( \phi \right) - s\left( {\phi_{r} } \right)} \right] $$

where *s*(*ϕ*) and *s*(*ϕ*_*r*_) are spatially averaged array responses determined with closed shutter at actual and reference operating conditions, respectively. Substituting Eq. 3 into Eq. 4 and arranging the resulting equation we obtain the final formula of the proposed NUC algorithm in the form

5 $$ y_{i} \left( \phi \right) = \left[ {x_{i} \left( \phi \right) - s_{i} \left( \phi \right)} \right]g_{i} + o_{i} + s\left( \phi \right) $$

where *g*_*i*_ and *o*_*i*_ are fixed gain and offset correction coefficients, respectively. The pixel gain correction coefficient, *g*_*i*_, is determined according to Eq. 2 while the fixed pixel offset correction coefficient, *o*_*i*_, is given by

6 $$ o_{i} = \left[ {s_{i} \left( {\phi_{r} } \right) - x_{i} \left( {\phi_{1} } \right)} \right]g_{i} + x\left( {\phi_{1} } \right) - s\left( {\phi_{r} } \right) $$

**Fig. 1:**
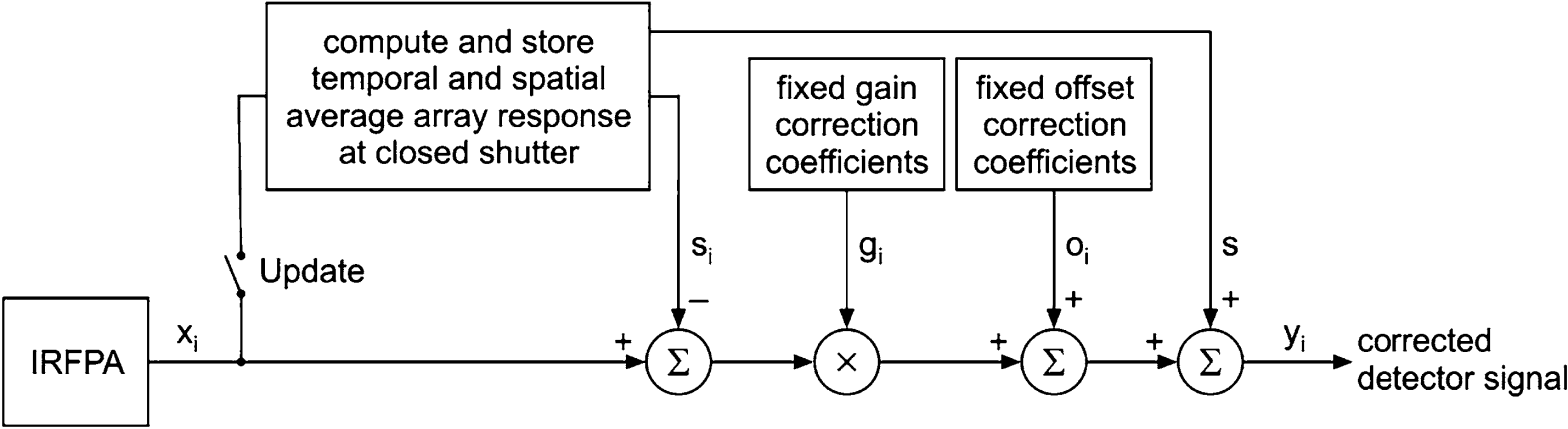
Block diagram of the proposed NUC algorithm

The evaluated fixed gain and offset correction coefficients are valid at the given operating conditions and the thermal scene dynamic range. They are stored in non-volatile memory of the user readout system and applied to the IRFPA output signal correction. In order to update pixel offset correction coefficients the user has to determine temporal and spatial average array response at closed shutter and then use their according to Eq. 5. A simplified block diagram of the proposed NUC algorithm is shown in Fig. 1. It is assumed that the IRFPA provides a digital output in otherwise the suitable analog-to-digital converter (ADC) must be applied. When the switch *Update* depicted in Fig. 1 is closed then the temporal average IRFPA response at closed shutter is calculated from the *N* consecutive image frames. In many cases the averaging of 50–100 image frames is sufficient to reduce the influence of pixel temporal noise. Additionally, if *N* is equal to the power of two we can obtain the average value by simple operations of data accumulating and bit shifting. However some external memory is needed to store acquired image data and intermediate results of the averaging process. Using a modern field-programmable gate array (FPGA) device that includes hardware multipliers and adders, it is easy to obtain a real-time implementation of the proposed NUC algorithm.

## Test results

The LWIR 384 × 288 amorphous silicon microbolometer IRFPA was taken under the tests. It features a high uniform pixel responsivity with the mean value of 7.2 mV/K (at FPA 303 K, F/1, 50 Hz) and the standard deviation of 1.1 % (Trouilleau et al. 2005). The IRFPA readout system was developed based on the useful Altera FPGA development kit. The special proxy board including: IRFPA bias circuitry, 14-bit ADC for video output signal and TEC controller was also designed. The detector integration time and frame rate at full pixel resolution were equal to 59.3 µs and 53 Hz, respectively. The Umicore 60 mm F/1.1 Gasir Standard Lens for LWIR range was applied to the IRFPA. For tests home-produced extended area blackbodies were employed as the IR references. The performance parameters of the conventional and modified TPC algorithm are listed in Tables 1 and 2, respectively. The calibration points for both NUC algorithms were flat-field images of 17.5 and 39.5 °C blackbodies, respectively. All image data for calibration was taken as 100-frame sequences averaged afterwards. The fixed gain and offset correction coefficients were calculated according to Eqs. 2 and 6, respectively. For testing NUC algorithms the second 100-frame image sequences of shutter and four blackbodies including calibration points were collected about 20 min after the basic calibration. Then nonuniformity correction on the new blackbody image sequences was performed with updated pixel offset correction coefficients according to Eqs. 1 and 5, respectively. To assess NUC algorithm efficiency noise components existing in the blackbody image after correction are estimated. Total noise in the image is defined by (Mooney et al. 1989)

7 $$ \sigma^{2} = \sigma_{t}^{2} + \sigma_{s}^{2} $$

which is the sum in quadrature of the temporal, *σ*_*t*_, and spatial noise, *σ*_*s*_. It is estimated by the standard deviation of pixel values in the flat-field image. The temporal noise is calculated as the mean standard deviation of pixel response in the array on uniform blackbody radiance registered in time, typically at 50 image frames. Then the spatial noise value is evaluated according to Eq. 7. The estimated values of noise components occurring in the single IRFPA response on uniform blackbody radiance after NUC correction are listed in Tables 1 and 2, respectively. The statistics of IRFPA response are stated in ADU (ADC units). The experimental results show that the proposed modification of TPC algorithm provides the improvement of FPN compensation for tested IRFPA sensor by factor of 2.9 at 28.5 °C flat-field image.

**Table 1:** Performance of the conventional TPC algorithm

Blackbody temperature (°C)	*m* (ADU)	*σ* (ADU)	*σ*/*m* (%)	*σ*_*t*_ (ADU)	*σ*_*s*_ (ADU)
17.5	3976.1	13.6	0.34	1.7	13.5
23.5	4101.7	13.6	0.33	1.8	13.5
28.5	4186.2	14.2	0.34	1.8	14.1
39.5	4450.8	13.9	0.31	1.8	13.8

**Table 2:** Performance of the modified TPC algorithm

Blackbody temperature (°C)	*m* (ADU)	*σ* (ADU)	*σ*/*m* (%)	*σ*_*t*_ (ADU)	*σ*_*s*_ (ADU)
17.5	3976.0	4.0	0.10	1.7	3.6
23.5	4101.6	4.2	0.10	1.8	3.8
28.5	4186.1	5.2	0.12	1.8	4.9
39.5	4450.7	4.7	0.11	1.8	4.3

**Fig. 2:**
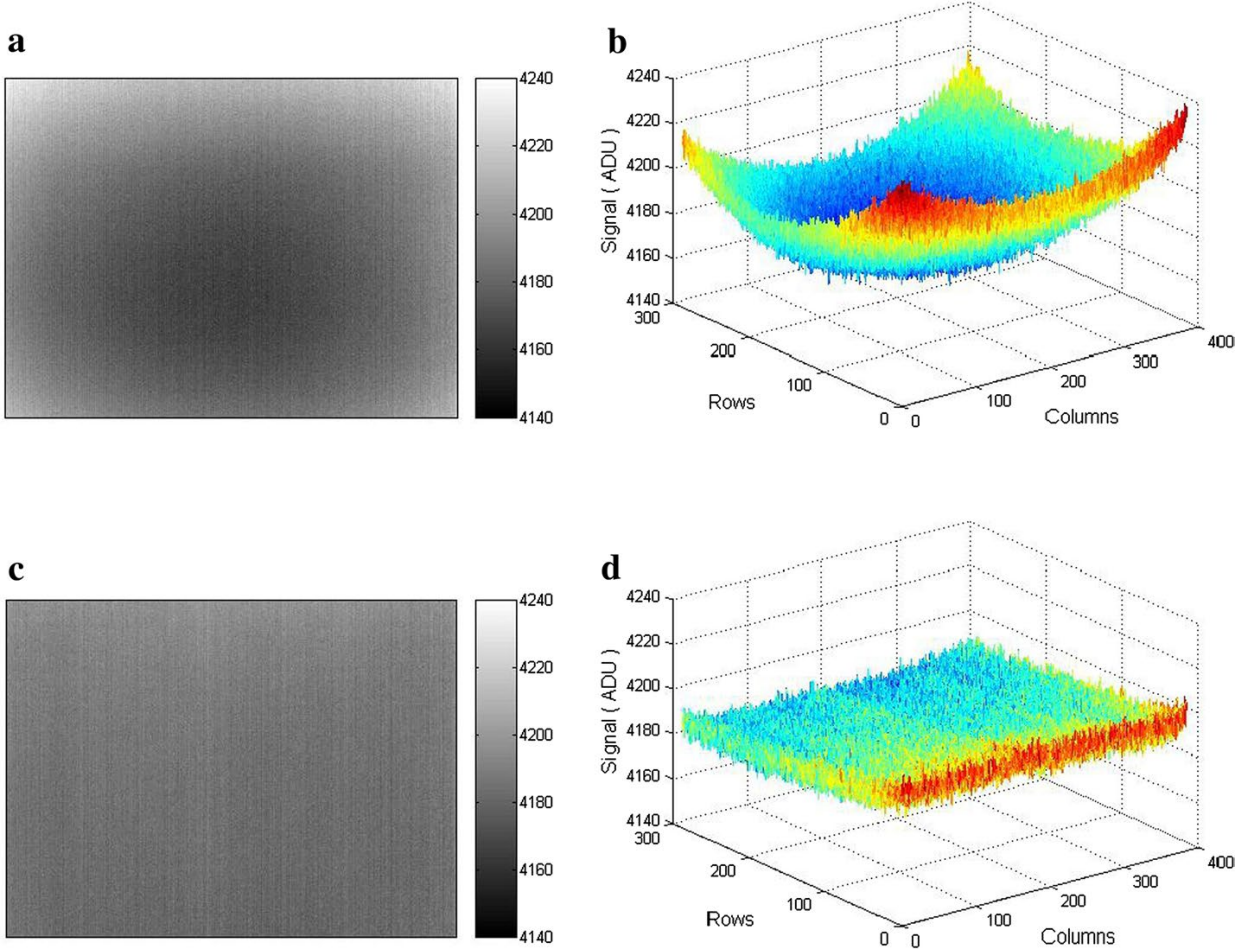
NUC results for microbolometer IRFPA response on 28.5 °C blackbody: **a** output image with optics shading effect (*σ*/*m* = 0.34 %), **b** 3D plot of IRFPA response (**a**), **c** output image after using the proposed NUC algorithm (*σ*/*m* = 0.12 %), **d** 3D plot of IRFPA response (**c**). Output span is 100 ADU. Calibration points for NUC are IRFPA responses on 17.5 and 39.5 °C blackbodies, respectively. Ambient temperature is 17.5 °C

The two-point NUC results for microbolometer IRFPA response on 28.5 °C blackbody are shown in Fig. 2. Figure 2a depicts the output image obtained after pixel offset drift compensation using a shutter according to the one-point calibration scheme. The output image includes a characteristic optics shading effect. For comparison Fig. 2c presents the correction result obtained after using the proposed NUC algorithm. The resulting image is most uniform than previous one and the residual pixel response nonuniformity is equal to 0.12 % (*σ*/*m*). In both cases the shutter was activated about 20 min after performing of two-point calibration procedure.

**Fig. 3:**
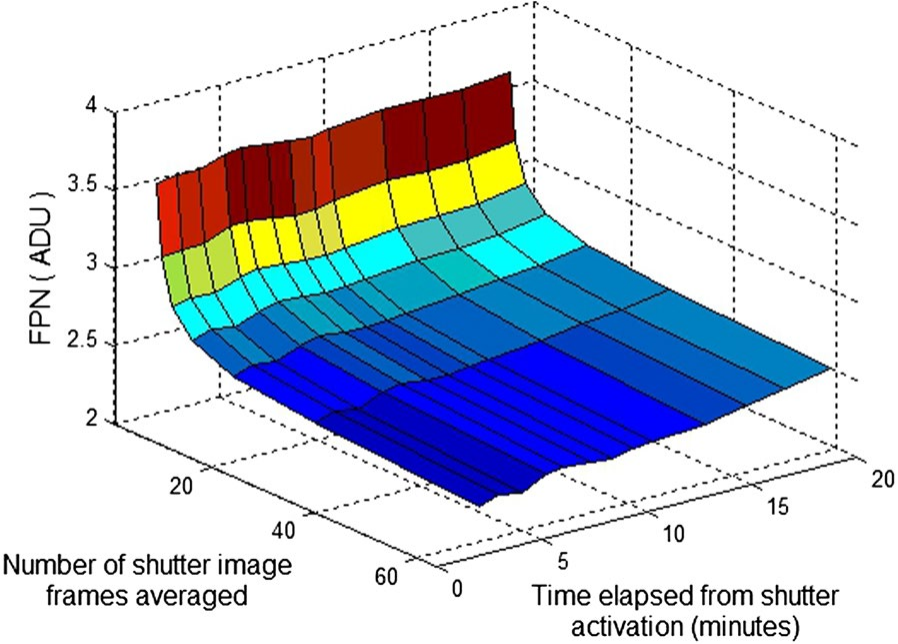
FPN value as a function of number of shutter image frames used in correction and time elapsed from shutter activation to correction of 30 °C blackbody image by the proposed NUC algorithm

**Fig. 4:**
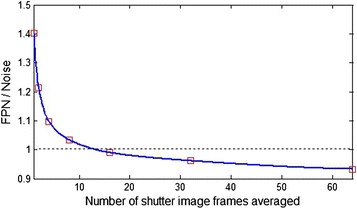
FPN to temporal noise ratio versus number of shutter image frames used in correction of 30 °C blackbody image by the proposed NUC algorithm. Time elapsed from shutter activation is about 2 min

**Fig. 5:**
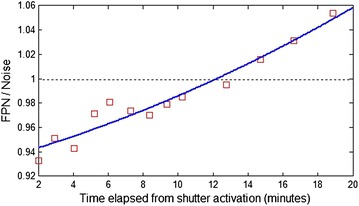
FPN to temporal noise ratio versus time elapsed from shutter activation to correction of 30 °C blackbody image by the proposed NUC algorithm. The 64-frame average of shutter image sequence is used in correction

A study was performed to determine the number of shutter image frames required to update offset correction coefficients in the proposed NUC algorithm. The new data set was collected from the 384 × 288 microbolometer IRFPA. The 64-frame image sequences of reference shutter and two blackbodies of 20 and 40 °C temperature were collected for calibration. To calculate fixed NUC coefficients 64-frame averages of the image sequences were taken. The calibration points were flat-field images of 20 and 40 °C blackbody, respectively. To investigate if the temporal averaging relaxes the frequency of calibration, the second data set was collected from the microbolometer IRFPA. At first 64-frame image sequence of actual shutter was taken and after that 64-frame flat-field images of 30 °C blackbody were registered within time of 20 min with step of about 2 min. The shutter image sequence was temporally averaged with the number of frames equal to power of two. Finally the nonuniformity correction on each of the 30 °C flat-field image sequences was performed according to Eq. 5 and the temporal and spatial noise value in the corrected image were estimated. The results of this analysis are shown in Figs. 3, 4 and 5. Figure 3 depicts a surface plot of the FPN value as a function of number of shutter image frames used in correction and time elapsed from shutter activation to correction of 30 °C flat-field image by the proposed NUC algorithm. In considered example the FPN value exponentially decreases with increase of the number of shutter image frames temporally averaged without the need of frequent calibration. The FPN to temporal noise ratio versus the number of shutter image frames used in correction of 30 °C flat-field image is shown in Fig. 4. As we can see the FPN value decreases below temporal noise level just at above 16 shutter image frames averaged to update pixel offset correction coefficients in the proposed NUC algorithm. Figure 5 depicts the FPN to temporal noise ratio as a function of time elapsed from shutter activation to correction of 30 °C flat-field image by the proposed NUC algorithm. It is seen that the FPN value is still below temporal noise level within 12 min from shutter activation using 64-frame average of shutter image sequence to correction. In considered example the temporal noise did not exceed value of 2.5 ADU at 50 image frames.

## Conclusions

The modified two-point NUC algorithm enabling pixel offset correction coefficients update by the proper usage of temporally averaged IRFPA response determined with closed shutter is proposed. The use of pixel response change determined by the shutter at the actual IRFPA operating conditions in relation to the reference ones provides good detector offset temporal drift compensation and optics shading effect removing as well. The performed tests with microbolometer IRFPA confirm a high efficiency of the proposed NUC algorithm that is easy to implement by hardware too. In case of the thermal imager operating within wide ambient temperature range, the several fixed gain and offset correction coefficients tables are required.
